# The Impact of Gelatin on the Pharmaceutical Characteristics of Fucoidan Microspheres with Posaconazole

**DOI:** 10.3390/ma14154087

**Published:** 2021-07-22

**Authors:** Marta Szekalska, Aleksandra Citkowska, Magdalena Wróblewska, Katarzyna Winnicka

**Affiliations:** Department of Pharmaceutical Technology, Medical University of Białystok, Mickiewicza 2c, 15-222 Białystok, Poland; citkowska.aleksandra@gmail.com (A.C.); magdalena.wroblewska@umb.edu.pl (M.W.)

**Keywords:** microparticles, fucoidan, gelatin, spray drying, antifungal activity

## Abstract

Fungal infections and invasive mycoses, despite the continuous medicine progress, are an important globally therapeutic problem. Multicompartment dosage formulations (e.g., microparticles) ensure a short drug diffusion way and high surface area of drug release, which as a consequence can provide improvement of therapeutic efficiency compared to the traditional drug dosage forms. As fucoidan is promising component with wide biological activity *per se*, the aim of this study was to prepare fucospheres (fucoidan microparticles) and fucoidan/gelatin microparticles with posaconazole using the one-step spray-drying technique. Pharmaceutical properties of designed fucospheres and the impact of the gelatin addition on their characteristics were evaluated. An important stage of this research was in vitro evaluation of antifungal activity of developed microparticles using different *Candida* species. It was observed that gelatin presence in microparticles significantly improved swelling capacity and mucoadhesiveness, and provided a sustained POS release. Furthermore, it was shown that gelatin addition enhanced antifungal activity of microparticles against tested *Candida* spp. strains. Microparticles formulation GF6, prepared by the spray drying of 20% fucoidan, 5% gelatin and 10% Posaconazole, were characterized by optimal mucoadhesive properties, high drug loading and the most sustained drug release (after 8 h 65.34 ± 4.10% and 33.81 ± 5.58% of posaconazole was dissolved in simulated vaginal fluid pH 4.2 or 0.1 M HCl pH 1.2, respectively).

## 1. Introduction

Fungal infections are still a serious problem in many, even highly developed countries. Among the various types of fungal infections, candidiasis is the most commonly diagnosed [1]. Species of *Candida* are opportunistic fungi, which are a component of the human flora of the digestive tract, vagina or skin, and they do not cause any disease symptoms in most people. However, in the case of reduced immunity, *Candida* species can induce dermatological and systemic infections [2,3]. The treatment of candidiasis is long-drawn and difficult, which is related to growing fungi resistance. Therefore, the search for new compounds, new combinations of drugs and novel drug delivery systems enabling effective and safe therapy are still a current topic.

Fucoidan (FUC) is a polysaccharide rich in sulfate groups isolated from brown algae. Its sources are also marine invertebrates such as sea urchins eggs and sea cucumber tissues [4]. The basic unit of the FUC structure is L-fucose, but other monosaccharides such as xylose, galactose, mannose, glucose, rhamnose or uronic acid are also included. The polysaccharide chain is most often formed by α1-3 and/or α1-4-linkages between L-fucose molecules. The sulfate groups characteristic for FUC are located at the C2, C-3 or C4 positions [5]. The growing interest in FUC utilization is due to its wide biological activity *per se* [6]—it is a promising component of antibacterial [7], anticancer [8], antifungal and antiviral therapies [9]. The molecular weight, the number of sulfate groups and the place of their substitution in FUC molecules significantly affects its activity [10]. This in turn is largely related to the species, from which FUC is obtained, the conditions of growth environment, time of harvest and the type of extraction method [5,11]. The possibility of FUC exploitation in the pharmacy and biomedicine also results from its safety, biocompatibility and biodegradability, which was confirmed by the Food and Drug Administration (FDA) decision to include FUC in the GRAS (Generally Recognized As Safe) category [12].

Gelatin (GEL) is a compound obtained from collagen by acid or alkaline hydrolysis or by degradation under the influence of temperature or enzymes. A characteristic component in the GEL structure, responsible for the ability to cell adhesion, is the aminoacid sequence Arg-Gly-Asp (RGD) [13]. This amphiphilic polymer is biocompatible, biodegradable and safe, categorized by the FDA as GRAS [12]. Moreover, the low cost of acquisition, as well as the ease of modification of its chemical structure, contribute to the widespread use of GEL in the pharmaceutical technology [14].

Posaconazole (POS) is a safe and well-tolerated second generation triazole derivative used both in the prevention and treatment of invasive fungal infections [15]. Broad antifungal activity of POS is due to its ability to inhibit lanosterol 14α-demethylase—enzyme involved in egosterol synthesis, which leads to the loss of cell membrane stability. POS is effective against *Candida*, *Aspergillus*, *Fusarium* and *Zygomycetes* [16]. The Biopharmaceutics Classification System (BCS) includes POS to the second group, which is characterized by high permeability, but limited solubility. POS absorption is influenced by many factors, including pH of the environment, the meal consumed and other medications used, as well as the strength and number of POS doses [17].

Microparticles represent multicompartment drug delivery systems, in which the active substance can be dissolved or suspended in a polymer matrix. They can constitute not only solid forms of the drug, but can also be utilized in semi-solid or liquid formulations providing favorable pharmacokinetic profiles and improved drug bioavailability [18,19]. The commonly techniques used to prepare microparticles are coacervation, emulsification/solidification and solvent extrusion, which are characterized by many disadvantages such as the difficulty in controlling the process, relatively low drug encapsulation and the necessity of organic solvents usage. However, there are many innovative and new approaches like spray drying and its modifications, supercritical CO_2_ assisted electrospray, membrane emulsification, ultra-fine particle processing system and self-healing encapsulation, which are developed for more efficient and precise microparticles preparation [20,21]. Therefore, the objective of this study was to prepare fucoidan microparticles (fucospheres) and fucoidan/gelatin microparticles with POS. To create microparticles, the spray drying technique—a one-step method useful to formulate spherical particles was applied. In the next step pharmaceutical properties of formulated microparticles and the impact of the GEL addition on the properties of designed microparticles was evaluated. Additionally, the swelling and mucoadhesive ability were tested. An important stage of this research was an in vitro evaluation of antifungal activity of developed microparticles using different *Candida* species.

## 2. Materials and Methods

### 2.1. Materials

FUC from *Laminaria japonica* with molecular weight 10.5 kDa was purchased from Weihai Century Biocom Seaweed Co. (Shandong, China). Content of fucose and sulphate groups was determined at a level higher than 27%. GEL from bovine skin type B and Tween 80 was received from Sigma Aldrich (St. Louis, MO, USA). POS was attained from Kerui Biotechnology Co. Ltd (Xi’an, China). Acetonitrile and methanol were provided by Merck (Darmstadt, Germany). Water was distilled and passed through the Milli-Q Reagent Water System (Billerica, MA, USA). By dissolving in 1 L of water 5 g glucose, 2 g lactic acid, 3.51 g natrium chloride, 1.40 g potassium hydroxide, 1.0 g acetic acid, 0.4 g urea, 0.222 g calcium hydroxide, 0.16 glycerol and 0.018 g bovine albumin, simulated vaginal fluid (SVF, pH = 4.2) was prepared [22]. Sabouraud dextrose agar (SDA) and stock cultures of *Candida albicans* ATCC^®^ 10231, *Candida krusei* ATCC^®^ 6528, *Candida parapsilosis* ATCC^®^ 22019 from American Type Culture Collection were purchased from Biomaxima (Lublin, Poland). Cellulose acetate membrane filters (0.45 µm) were provided by Millipore (Billerica, MA, USA), and nylon membrane filters (0.45 µm) were received from Alchem (Toruń, Poland). Porcine vaginal mucosa and stomach mucosa was procured from the veterinary service of a local abattoir (Turośń Kościelna, Poland). Commercially available tablets with fluconazole (Fluconazole Polfarmex 50 mg; Polfarmex S.A. batch no.: 011117) were purchased locally (Polfarmex S.A., Kutno, Poland), and used as a control in the agar diffusion test.

### 2.2. Preparation of Microparticles

Fucoidan microparticles were obtained by the spray drying of aqueous FUC solutions using Mini Spray Dryer B-290 (Büchi, Switzerland) (Table 1). To obtain FUC/GEL microparticles, 5% GEL solution at 40 °C was added dropwise, using a magnetic stirrer. The experimental parameters of the spray drying process were optimized and set as follows: inlet temperature 122 °C, outlet temperature 62 °C, pressure 60 mm Hg, aspirator blower capacity 100% and feed rate 2.1 mL/min.

### 2.3. Shape and Size

To characterize morphology and shape of designed microparticles, a scanning electron microscope (SEM) (Hitachi S4200, Tokyo, Japan) was utilized. All microparticles were also observed by using an optical microscope equipped with a camera (Motic BA 400, Moticon, Wetzlar, Germany) under 40× magnification and particles size was analyzed.

### 2.4. Evaluation of POS Loading, Encapsulation Efficiency and Production Yield

To determine POS loading, 10 mg of microparticles was dissolved in 1 mL of distilled water and agitated for 1 h at 75 rpm in a water bath (37 ± 1 °C). Then, 9 mL of methanol was added and agitated for 1 h. 0.5 mL of filtrated solution was mixed with 9.5 mL of phase (acetonitrile: methanol: water 60:20:20, v/v) and examined by HPLC technique using Agilent Technologies 1200 system (Agilent, Waldbronn, Germany) equipped with Poroshell 120 EC-C18 2.7 µM ODS 4.6 mm × 150 mm, 2.7 µm column (Agilent, Waldbronn, Germany). The mobile phase was applied with the flow rate 0.5 mL/min. UV detection was achieved at a wavelength of 280 nm [23]. POS retention time was recorded at 5.5 min. Standard calibration curve was linear over the range of 1–100 µg/mL with the correlation coefficient (R^2^) 0.999.

Drug loading (L) was calculated from the expression

L = Q_m_/W_m_ × 100 (1)

where Q_m_—drug encapsulated in the microparticles, and W_m_—microparticles weight.

The mean drug encapsulation efficiency (EE) was determined by using the following formula

EE = Q_a_/Q_t_ × 100, (2)

where Q_a_ is the actual drug content and Q_t_ is the theoretical drug content. 

Yield of production (Y) was calculated using the equation

Y = W_m_/W_t_ × 100, (3)

where W_m_—weight of microspheres, W_t_—theoretical weight of drug and polymer [24].

### 2.5. Swelling Properties

To assess the swelling properties, 100 mg of microparticles were placed in a tube containing 5 mL of 0.1 M HCl or SVF. After a set period of time, the medium was removed and the swollen particles were weighed. The swelling capacity was expressed by swelling ratio (SR) calculated based on the expression

SR = (W_s_ − W_0_)/W_0_, (4)

where W_0_ is the microparticles initial weight and W_S_ is the weight of swollen microparticles [24].

### 2.6. Mucoadhesive Properties

Determination of the mucoadhesiveness was performed using TA.XT. Plus Texture Analyser (Version 6.1.1.0, Stable Micro Systems Ltd., Godalming, Surrey, UK). As mucoadhesive materials, porcine vaginal and stomach mucosa were used. After moisturizing with 50 µL of SVF or 0.1 M HCl, 100 mg of microparticles were exposed to 1 N contact force for 60 s. Pre-test speed, test speed and post-test speed were 0.5, 0.1 and 0.1 mm/s, respectively. Tests were conducted at 37 °C ± 1 °C. Maximum detachment force (F_max_) was registered by Texture Exponent 32 software and the work of mucoadhesion (W_ad_) was calculated from the area under the force versus distance curve.

### 2.7. In Vitro POS Release

Dissolution basket apparatus (Erweka Dissolution Tester Type DT 600HH, Heusenstamm, Germany) was used for the POS release test. Microparticles were suspended in 500 mL of SVF (pH 4.2) with addition of 2% Tween 80 or 0.1 M HCl (pH 1.2) to obtain sink conditions and stirred at 100 rpm at 37 ± 1 °C for 24 h. Samples were taken at the following time points: 0.5, 1, 2, 3, 4, 5, 8 and 24 h, and the medium volume was supplemented with an equivalent amount of fresh acceptor fluid. Analysis was performed by using spectrophotometer GENESYS 10S UV-Vis (Thermo Scientific, Waltham, MA, USA) at 280 nm (for SVF with 2% Tween 80) or 260 nm (for 0.1 M HCl).

### 2.8. Mathematical Modeling of the POS Release Profile 

To explain the drug release mechanism, data obtained from POS release tests were studied under different mathematical models [25].

Zero order kinetic:

F = k × t(5)

First order kinetic:

lnF = k × t(6)

Higuchi model:

F = kt^1/2^ (7)

Korsmeyer-Peppas model:

F = kt^n^ (8)

Hixson-Crowell model:

1 − (1 − F)^1/3^ = kt (9)

where F—the fraction of released drug, k—the constant connected with release, and t—the time.

### 2.9. Differential Scanning Calorimetry (DSC)

DSC analysis of unprocessed FUC, GEL and POS-loaded formulations with the higher drug loading (F4, GF4) and placebo formulations (F1, GF1) was accomplished using an automatic thermal analyzer system (DSC TEQ2000, TA Instruments, New Castle, DE, USA). Each sample was precisely weighed (5 mg) and then placed in a sealed aluminum pan. An empty pan sealed was used as a reference. As standard for temperature calibrations, indium and zinc were used. Samples were heated in the range from 25 °C to 300 °C at a scanning rate of 10 °C/min under a nitrogen flow of 20 mL/min.

### 2.10. Antifungal Activity

To evaluate the antifungal activity of the designed microparticles, the agar diffusion method was used. Petri dishes containing Sabouraud’s dextrose agar were seeded with 50 µL of the fungal inoculums prepared using sterile 0.9% NaCl solution, with final density 5 × 10^6^ CFU/mL (corresponding to 0.5 in McFarland scale). After 15 min of drying at room temperature, wells with a diameter of 5 mm were cut out in agar plates, into which 10 mg of the microparticles of the tested formulations were placed. As controls, 50 µL of the solution obtained by dissolving POS in DMSO (corresponding to 4 mg of POS), 10 mg of FUC and GEL powders were used. Plates were incubated at 37 ± 0.1 °C for 24 and 48 h. After this time the growth inhibition zone was measured using a caliper (Mitutoyo, Kawasaki, Japan) with an accuracy of 0.1 mm.

### 2.11. Statistics

The obtained results are presented at the mean ± standard deviation (SD) based on three or six independent experiments (n). Data were assessed by Statistica 10.0 (StatSoft, Tulsa, OK, USA) using one-way analysis of variance (ANOVA) or a Kruskal-Wallis test. Obtained results were presented as the mean and standard deviation.

## 3. Results and Discussion

### 3.1. Microparticles Characteristics

In the last decades, despite the continuous medicine progress, fungal infections and invasive mycoses still constitute an important globally therapeutic problem. Low effectiveness of treatment is associated with the limited numbers of available antifungal compounds together with existence of many pathogenic drug-resistant fungi species. Therefore, new antifungal therapies are still required [26]. Compared to the traditional one-unit forms, multicompartment formulations (e.g., microparticles) ensure short drug diffusion way and high surface area of drug release, which can provide the improvement of therapeutic efficiency [18,27].

**Figure 1:**
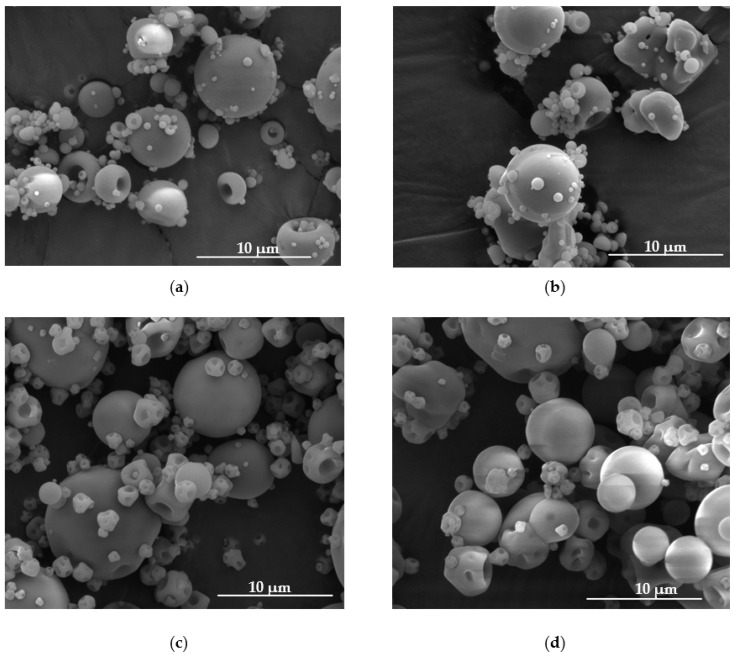
Representative images of microparticles formulation F1 (**a**), F4 (**b**), GF1 (**c**) and GF4 (**d**) under magnification ×10,000.

Spray drying is a method often used in chemical, food, biotechnological and pharmaceutical industries and possesses many advantages. This one-step continuous process allows the liquid to be converted into a dry powder in a relatively simple way and obtaining a product with specific properties, which depend on many variables [28]. The morphology of microparticles was evaluated by SEM analysis, and is presented in Figure 1.

It was observed that one-step spray drying process might be successfully used both to receive FUC and FUC/GEL microparticles. Fucospheres placebo (F1) possess uniform and smooth surface with characteristic concave depressions (Figure 1a). FUC/GEL microparticles placebo (Figure 1c) were mostly spherical, and only few irregularly shaped microparticles with a pinhole were observed. This fact was also observed by Panizzon et al., who prepared gelatin microspheres with soy isoflavones by the spray drying technique [29]. Interestingly, POS presence and GEL addition did not significantly affect the particle size (Figure 1, Table 2). Additionally, microparticles with POS, compared to the placebo formulations, were characterized by a spherical shape with smooth and poreless surfaces.

The quality assessment of prepared microparticles includes analysis of production yield, moisture content, particle size, POS percent loading and POS encapsulation efficiency, see Table 2. It was observed that the spray drying method allowed to obtain FUC and FUC/GEL microparticles placebo (formulations F1–F3 and GF1–GF3) and FUC and FUC/GEL microparticles with POS (formulations F4–F6 and GF4–GF6) with relatively high production yield as illustrated in Table 2. The addition of GEL caused a significant (*p* < 0.05) decrease in the value of this parameter. One of factors affecting product characteristics and process performance is the viscosity of the dried solution. As the feed viscosity increases, the size of obtained particles also increases. Too-large particles are not completely dried, and they deposit on the chamber walls [29,30]. The result is lower process efficiency, which was observed in formulations obtained from FUC/GEL solutions (Table 2) possessing higher viscosity values than pure FUC (10% FUC solution viscosity was 18.8 mPa∙s, and 10% FUC solution with addition of 5% GEL—780.7 18.8 mPa∙s). It was also noted that FUC solutions viscosity affects microparticles size—microparticles diameter was ranged between 12.02 ± 5.14 µm and 17.91 ± 4.35 µm (Table 2). Higher feed viscosity leads to the formation of larger droplets, and as a consequence, particles obtained during the spray drying process were characterized by larger diameters. A comparable result was observed in work of Ogunjim et al. [31], where the spray drying of different chitosan solutions with low (0.5% *w/v*), medium (0.7% *w/v*) and high (0.9% *w/v*) concentrations significantly affected microspheres size.

The moisture content in spray-dried microparticles was relatively low and ranged between 5.19 ± 0.87% for formulation GF5 and 9.81 ± 2.84% for formulation F3.

A drug content within the particles is an important parameter for the dose administration. POS loading in the microspheres and encapsulation efficiency in formulations F4–F6 and GF4–GF6 are summarized in Table 2. The percent loading and encapsulation efficiency changed in a similar way. Both the increase in FUC concentration and the addition of GEL caused only a slight reduction of the POS loading (Table 2). The highest values of percent loading was obtained in the F4 formulation (46.16 ± 0.47%), and EE—in the formulation F6 (105.83 ± 8.17%). It was observed that EE values were decreased as a result of GEL addition. The combination of polymers (FUC and GEL) results in a decrease in the amount of free charges, which means that less of the drug can be bounded than when polymers were used individually. Similar results were obtained by Tejada et al. in the case of microparticles composed with chitosan, GEL and a mixture of these polymers. The EE of miconazole nitrate for formulations containing single polymer: chitosan or gelatin was 97.8% and 98.5%, respectively. However, the combination of these polymers resulted in a decrease in the EE value to 83.2% [32]. The lower EE might be also a result of POS adhesion to the walls of the drying chamber. Additionally, larger POS particles could be eroded during the spray drying process due to their high surface areas [30,33], and small particles of POS might be removed by the airflow to the filter collector. The similar result was observed in Pomázi et al.’s work, where meloxicam percent loading in mannitol-polyvinylpyrrolidone-based microcomposites was significantly higher (48.95% versus 27.74%) in comparison to the drying process without polyvinylpyrrolidone application [34].

### 3.2. Swelling and Mucoadhesion Ability

The swelling capacity of microparticles is a key element for both mucoadhesion and drug release process. Adhesive polymers enable extended contact of drug with the mucosa in the appliaction site as a result of mucoadhesion process. The first, most important step of mucoadhesion phenomenon is the polymer swelling after contact with moisture. Swellable polymer becomes more plastic through relaxation of the polymer chains, which increases the possibility of forming van der Waals or hydrogen bonds and enables their penetration through the mucus, and as a consequence—adhesion. Moreover, the process of drug release strictly depends on the ability of the polymer to swell [35].

**Figure 2:**
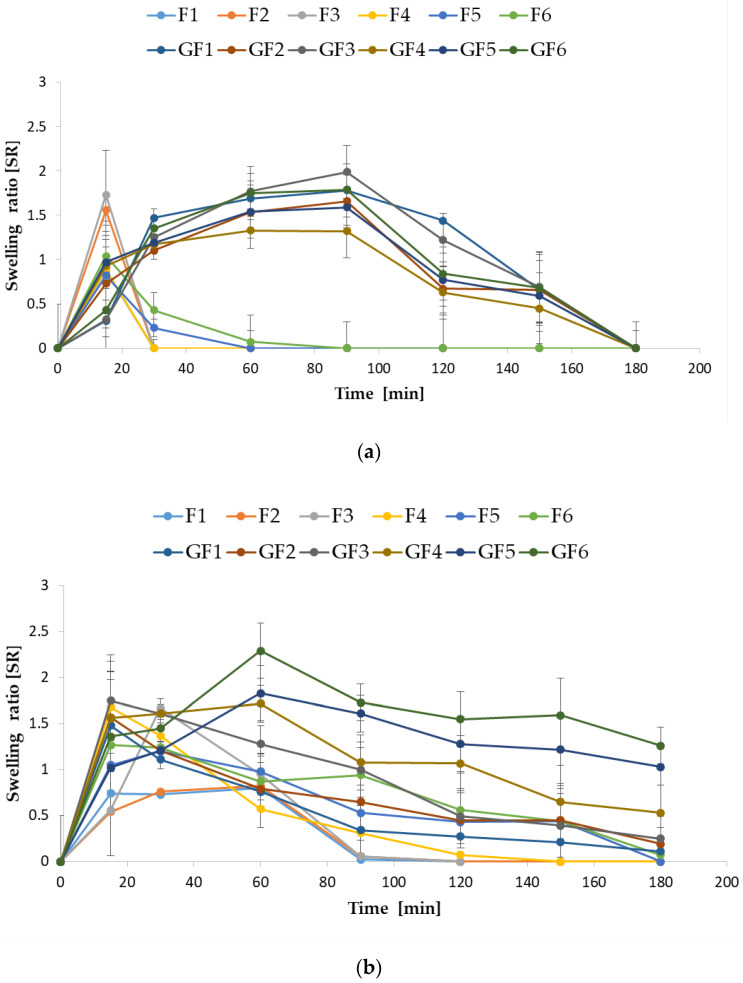
Swelling ratio of placebo (F1–F3, GF1–GF3) and POS-loaded (F4–F6, GF4–GF6) microparticles in (**a**) SVF at pH 4.2 and (**b**) 0.1 M HCl at pH 1.2 (mean ± SD, *n* = 3).

The swelling study was performed for all formulations using SVF (pH 4.2) and 0.1 M HCl (pH 1.2) to imitate vaginal and stomach environment, respectively (Figure 2a,b). The results obtained in both media showed that the higher FUC content in the composition resulted in better microparticle swelling ability. This is related with the fact that FUC solutions are characterized by linear viscosity increase [36]. It was also observed that higher pH (4.2) caused a decrease in swelling capacity, especially for formulations composed only from FUC. The same dependence between the degree of swelling and the medium pH was noted for FUC/chitosan beads (in pH 1.2 it was over 5, and in pH 6.0 it was less than 4) [37]. Lower swelling capacity was observed in the vaginal fluid (SVF, pH 4.2), which is related to the higher solubility of FUC in this medium. The SR graph of the fucospheres placed in SVF shows a sharp swelling peaks after 15 min with values from 0.88 ± 0.03 for F1 formulation to 1.93 ± 0.24 for F3 formulation and reaches zero value after 30 min. As FUC solutions are not highly viscous, it is not industrially used as thickening or gelling agent, as many other polysaccharides—only little gelling and film forming properties of FUC were described [38]. It was observed that GEL presence in microparticles matrix significantly increased their swelling capacity in comparison to microparticles containing only FUC. It was observed that the microparticles (GF1–GF3) swelled gradually in the vaginal fluid, reaching maximum values after 90 min (from 1.73. ± 0.23 in GF1 to 1.99 ± 0.12 in GF3). The addition of GEL, which solubility depends on temperature and which creates solutions with higher viscosity than FUC, leads to the increased stability of microparticles and the lower rate of water penetration into the gel during the swelling process. This might be attributed to a high volume of swollen microparticles, which can absorb a lot of solvent during the initial period of swelling [39]. The influence of GEL content on swelling capacity of scaffolds made of sodium alginate, GEL and FUC was also noted by Nguyen et al. They observed the highest SR values in scaffolds in which the GEL share was the largest [40]. The swelling capacity of films made of microfibrillated chitin and GEL prepared by Li et al. changed in similar way [41].

Although FUC is stable over a wide pH range (from 5.8 to 9.5), in the acidic medium acid hydrolysis might occur, and a decrease in the FUC solubility is observed [4]. This can be seen on the graph of swelling, where fucospheres placebo in 0.1 M HCl (pH 1.2) possessed the highest SR values after 30 min (from 0.73 ± 0.07 for formulation F1 to 1.67 ± 0.11 for F3). However, POS-loaded formulations were characterized by a higher SR and reached the highest values after 15 min (from 1.27 ± 0.12 for formulation F6 to 1.68 ± 0.21 for F4). Interestingly, when HCl was used, FUC/GEL microparticles placebo showed the maximum swelling after 15 min (SR value from 1.48 ± 0.29 for formulation GF1 to 1.75 ± 0.13 for formulation GF3), which slightly decreased during the experiment. However, FUC/GEL microparticles containing POS reached maximum SR value after 60 min (from 1.72 ± 0.07 for formulation GF4 to 2.29 ± 0.29 for formulation GF6) and their swelling remained almost at the same level until the end of the experiment (up to 180 min).

Mucoadhesive polymers applied in formulation significantly elongate the contact time of the dosage forms with the mucosa at the application place as a result of complex mechanism based on the formation of van der Waals forces or hydrogen, ionic and covalent bonds. Consequently, they might promote long-term drug release, as well as the increase in plasma drug concentration and higher drug bioavailability. When designing vaginal dosage forms, an important parameter is the favorable interaction with the mucosa that keeps the drug dosage form in place and shelters it from leakage. In turn, mucoadhesive formulation in oral delivery extends the residence time of the drug in the stomach, which is especially important for sparingly soluble substances with low bioavailability [42]. In this study, to imitate the in vivo environment, porcine vaginal and stomach mucosa as useful and valuable mucoadhesive adhesive layers were applied [42,43].

**Figure 3:**
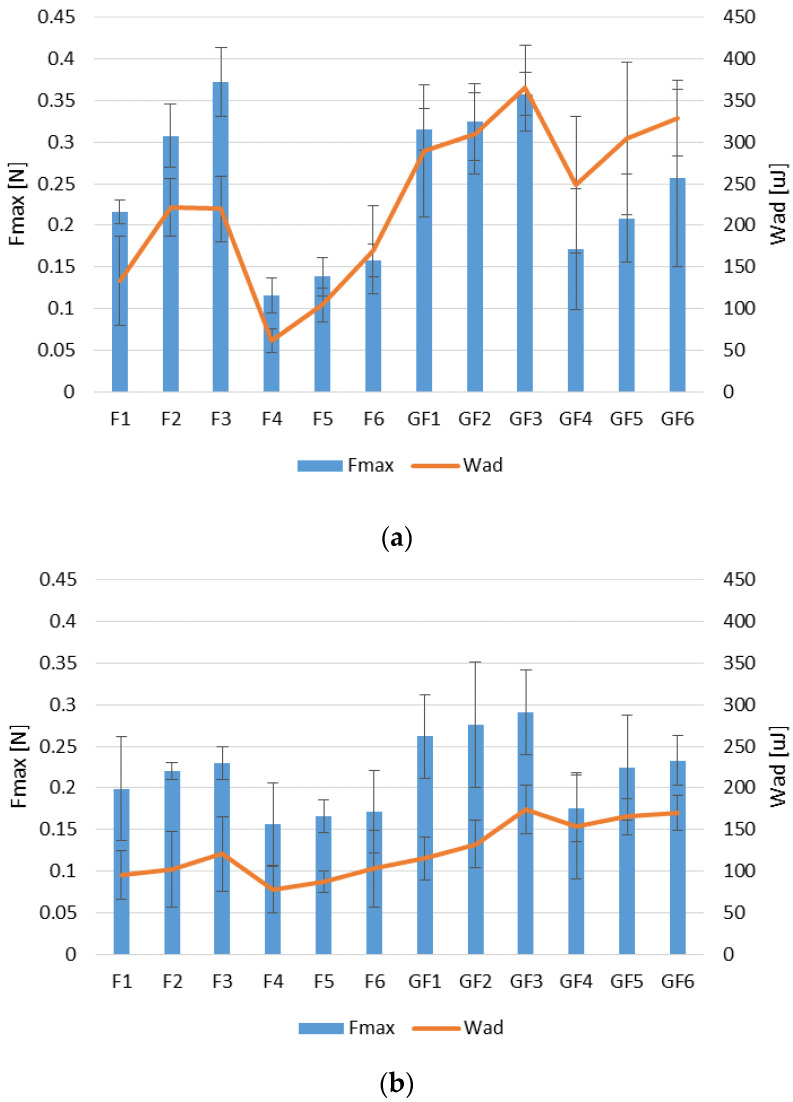
Mucoadhesiveness of fucospheres (F1–F6) and FUC/GEL microparticles (GF1–GF6) using (**a**) vaginal mucosa and (**b**) stomach mucosa expressed as detachment force (F_max_) and work of adhesion (W_ad_) (mean  ±  SD, *n* = 6).

It was observed that both fucospheres and FUC/GEL microparticles exhibited mucoadhesive properties (Figure 3). Studies using the vaginal and stomach mucosa have shown that mucoadhesive properties expressed as detachment force F_max_ (N) and the work of adhesion W_ad_ (μJ) increased with the increase of FUC concentration. Similar relation was also noted for chitosan and chitosan/FUC gels designed by Sezer et al.—formulations with FUC addition were characterized by the greater possibility of water absorption and higher swelling ratio than pure chitosan gels, and formulations containing the highest polymer concentrations (0.75% FUC and 2% chitosan) showed the greatest adhesion values [44].

The conducted research showed that the microparticles exhibited greater mucoadhesive properties when the vaginal environment was imitated. This is closely related to the good solubility of FUC in the medium (SVF, pH 4.2) used to wet the microparticles prior to testing and with the swelling ability in this medium.

GEL addition improved mucoadhesive properties of the designed microparticles. This phenomenon was especially visible in POS-loaded microparticles (F4–F6 compared to GF4–GF6), when vaginal mucosa was used (Figure 3a). Improvement of mucoadhesive properties through the addition of GEL was also observed in carrageenan films [45]. Microparticles with POS were characterized by lower mucoadhesiveness (with lower values of both F_max_ and W_ad_) compared to placebo formulations regardless of the adhesive layer used. This fact might be a consequence of blocking access of the polymers carboxyl groups to the mucous membrane by drug molecules. FUC mucoadhesive properties are not clearly explored, and there are only negligible studies concerning FUC adhesion mechanism. As FUC belongs to anionic polymers, it can be concluded that the mucoadhesion process is mainly related to the presence of the carboxyl groups, which form hydrogen bonds with the hydroxyl groups of mucin glycoproteins [42].

### 3.3. In Vitro POS Release

The in vitro release is a valid test utilized in the development and optimization of formulations, as well as to predict the behavior of the formulation in vivo. The in vitro release stage simulates drug availability for absorption. The first step of absorption is based on drug dissolution in the gastrointestinal or other fluid and then permeation across the membrane. Absorption of poorly water-soluble drugs is mainly drug dissolution dependent, and in vitro models are used to predict the dissolution substance from a solid drug delivery system and absorption from gastrointestinal tract [46]. Therefore, it is especially important to use mucoadhesive dosage forms, which by extending the contact time with the mucosa, prolong the residence time of the drug at the site of application, and as a result, retard drug dissolution and improve its bioavailability.

**Figure 4:**
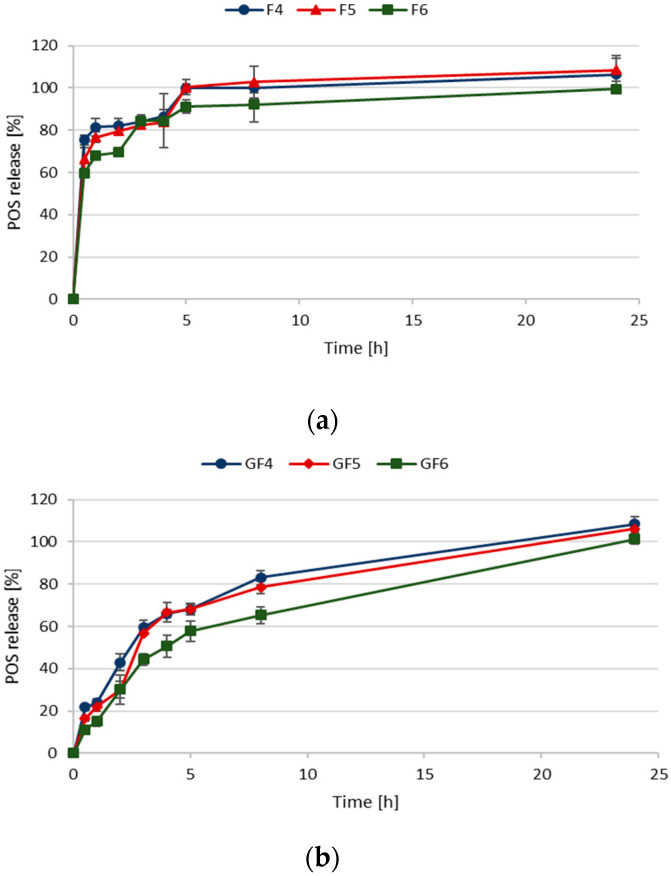
In vitro POS dissolution studies in SVF pH 4.2 from fucospheres (**a**) and FUC/GEL microparticles (**b**) (mean ± SD, *n* = 3).

**Figure 5:**
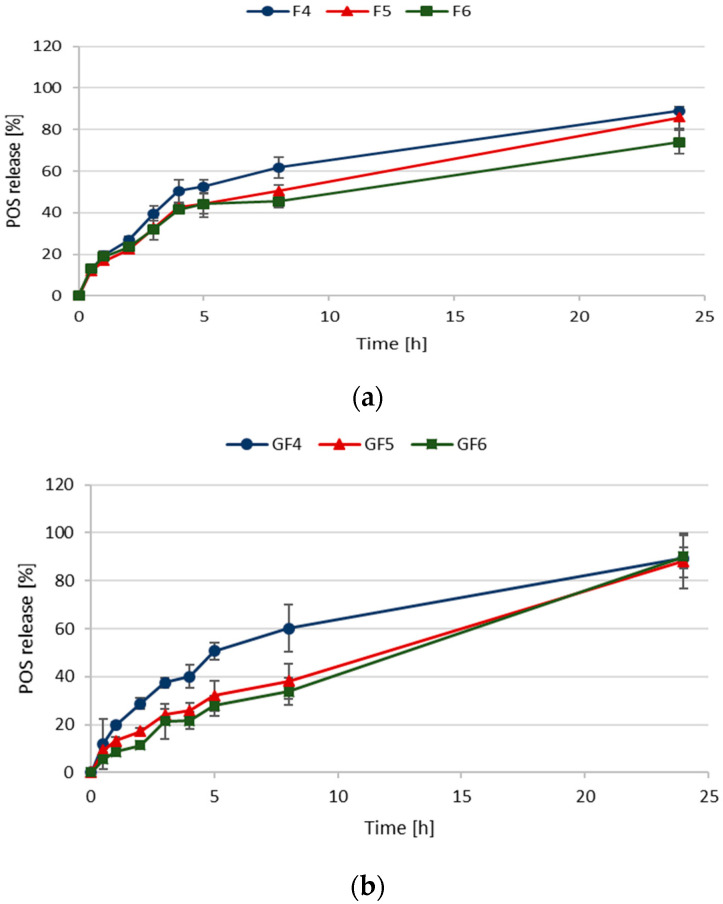
In vitro POS dissolution studies in 0.1 M HCl pH 1.2 from fucospheres (**a**) and FUC/GEL microparticles (**b**) (mean ± SD, *n* = 3).

Studies on POS release from designed microparticles in SVF (pH 4.2) used as an acceptor fluid showed that FUC concentration slightly affected the POS release profile (Figure 4a,b). Rapid drug burst occurred in F4–F6 formulations, so that after 0.5 h, from 59.77 ± 0.67% to 75.35 ± 2.33% of POS was released. 80% of POS from formulation F4 was released already after 1 h, but from formulation F6—after 3 h. The reason for this rapid POS release could be the high solubility of FUC in the medium [4]. A significant (*p* < 0.05) extension in POS release was observed in the formulations GF4–GF6, which is a result of GEL addition (Figure 4b). In formulations with lower FUC concentrations, no effect of FUC on drug release was observed (F4, F5; GF4, GF5). The most sustained POS release was observed in the case of formulation containing GEL and the highest FUC concentration (GF6)—after 0.5 h and 8 h, 59.77 ± 0.67% and 65.34 ± 4.10% of the active substance was dissolved in the medium, respectively. When 0.1 M HCl was used as the acceptor fluid, a significant extension of the POS release from all formulations was noted (Figure 5). In formulations with higher FUC concentration containing GEL (GF5 and GF6), prolonged POS release was observed. Formulation GF6 was characterized by the most extended drug profile—after 8 h, only 33.81 ± 5.58% of POS was released.

Generally, the swelling ratio of microparticles is a good indicator of drug release profile. Indeed, with the increase or decrease in the swelling ability, release of the drug occurs rapidly or the profile is prolonged. Observed SR values are in correlation with the POS release profiles—in the acidic medium microparticles swelled slower and longer than in the SVF (pH 4.2). As FUC is characterized by high solubility, FUC microparticles degraded faster than those containing GEL, therefore F4–F6 formulations provided faster POS release than GF4–GF6. This might be caused by gel layer around the GEL-containing microparticles, which hindered water influx, ensured slower microparticles degradation and prolonged drug release [40].

To predict POS release mechanism from microparticles, data received in the in vitro release study were modeled with different mathematical equations: zero-order and first-order kinetics, Higuchi, Korsmeyer–Peppas and Hixson–Crowell models (Table 3). It was observed that POS release kinetics was dependent on the medium applied in the performed tests. The data obtained from release test in SVF (pH 4.2) suggest that POS release was according to the zero order kinetics, which indicates that it is not concentration dependent. Meanwhile, when 0.1 M HCl (pH 1.2) was used in the study, POS release followed first-order kinetics, indicating that drug release rate was proportional to the amount remaining in the system. However, the obtained values of diffusion exponent (*n*) in the Korsmeyer-Peppas equation allow to predict the mechanism of drug release. In case of sphere shape microparticles, values of *n* ≤ 0.43 indicate that drug release is controlled by Fickian diffusion [25]. The obtained data from 0.09 to 0.21 in the SVF (pH 4.2) and from 0.19 to 0.28 in the 0.1 M HCl (pH 1.2) confirm diffusion as a mechanism of POS release. This fact was also confirmed by high values of R^2^ in the Highuchi model. Moreover, high values of R^2^ showed in the Hixson-Crowell model demonstrate that POS release was additionally controlled by the microparticles disintegration process. Thus, the obtained data indicate that POS release from fucospheres and FUC/GEL microparticles is complex, and includes erosion and diffusion mechanism. It is in the agreement with results published by Panizzon, who studied soy isoflavone release from spray-dried gelatin microspheres [29].

### 3.4. Differential Scanning Calorimetry (DSC)

**Figure 6:**
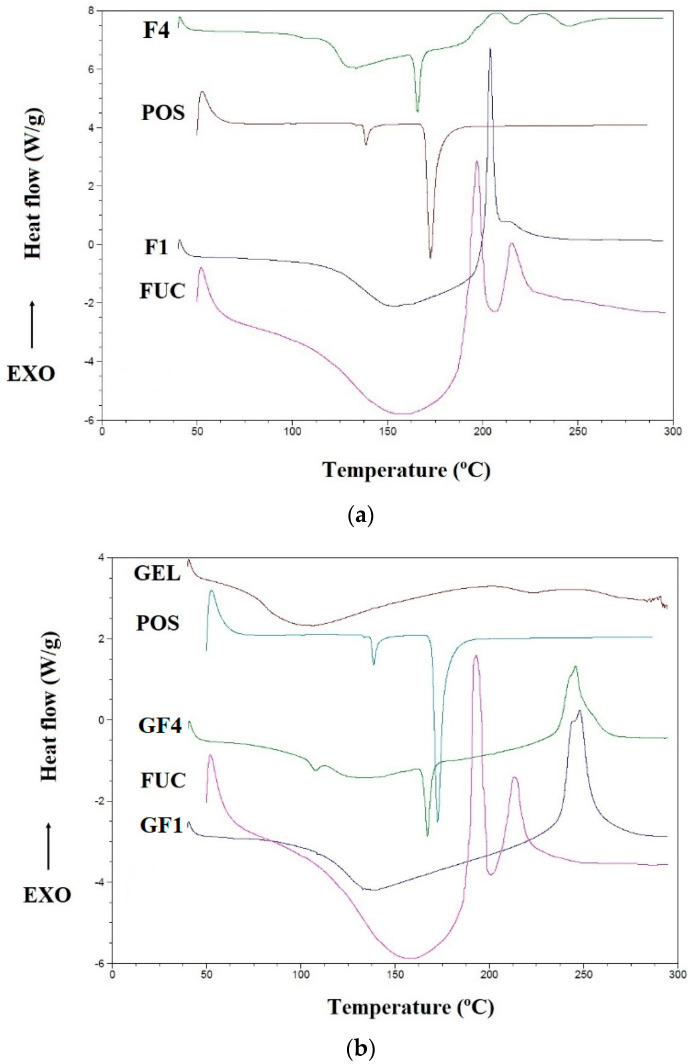
DSC thermograms of (**a**) POS, FUC, fucospheres F1, F4 and (**b**) POS, FUC, GEL and FUC/GEL microparticles GF1, GF4.

To estimate the thermal properties of POS and the polymers used, the DSC technique was applied. DSC thermograms provide information about possible interactions between drug and polymer through the analysis of parameters such as shift, appearance and disappearance of peaks, melting point and relative peak area [47]. Thermograms of the fucospheres, FUC/GEL microparticles and their components are shown in Figure 6. DSC curves of unprocessed FUC exhibited a broad endothermal peak between 125 °C and 187.5 °C, which indicates the loss of water content in the polymer. Additionally, two sharp exothermic peaks related to FUC decomposition at 190 °C and 215 °C were observed, which might be due to the polymer decomposition [48,49]. POS thermogram exhibits two sharp endothermic peaks. Peak at temperatures 138.73 °C corresponds to the loss of crystal water, at 172.70 °C—to intrinsic melting points of pure POS [50]. POS peak in formulation F4 demonstrated a slight decrease in the melting temperature (from 172.70 °C to 167.67 °C), which is probably a result of interactions between drug and the polymer, as well as the formation of a polymer matrix (Figure 6a) [51]. Lowering in POS melting point value in microspheres and disappearance a sharp peak at 138.73 °C might be due to its mixing with FUC, which lowered the purity of each component. Additionally, POS crystallinity was reduced and the drug converted into the amorphous form. In Figure 6b, DSC curves of GEL exhibited broad endothermal peaks between 75 °C and 125 °C, which demonstrate the loss of water content in the polymer. Thermogram of formulation GF4 demonstrated a sharp endothermic peak of POS at temperature at 167.52 °C, which was characterized by slight shift in relation to the peak of pure drug. The shift of the first POS peak from 138.73 °C to 112.5 °C, probably caused by mixing FUC with GEL, was also observed. In the formulations GF1 and GF4, a shift of broad endothermic peak of GEL was noted, which might be due to the loss of water by GEL. In the formulations GF1 and GF4, sharp exothermic peaks in the temperature 238 °C and 245 °C were observed, respectively.

### 3.5. Antifungal Activity

**Figure 7:**
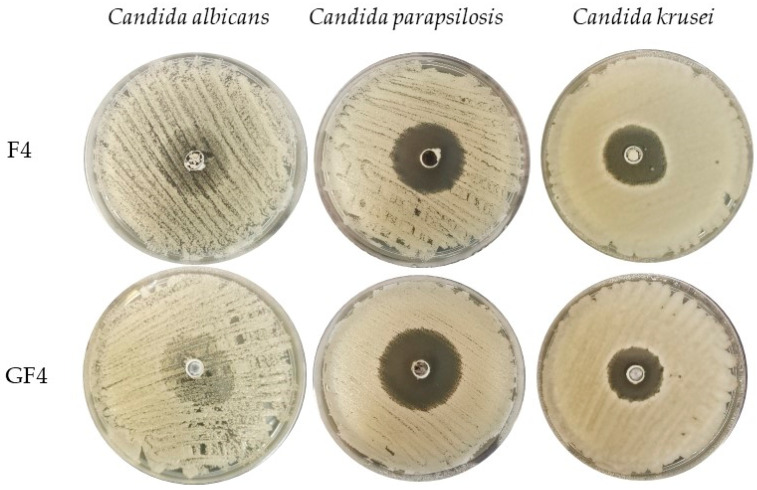
Representative images of zone inhibition in *Candida albicans*, *Candida parapsilosis* and *Candida krusei* strains (formulation F4 and GF4).

The obtained microparticles were evaluated for antifungal activity using one of the most popular methods—the agar diffusion test [52]. The inhibition zone was tested for three strains: *Candida albicans*, *Candida parapsilosis* and *Candida krusei* (Table 4, Figure 7). *Candida parapsilosis* turned out to be the most sensitive strain to the tested formulations, and the largest inhibitions of growth appeared in case of GF4 microparticles (43.2 ± 2.5 mm). Placebo microparticles with the addition of GEL (GF1–GF3) showed stronger antifungal activity against all strains compared to those consisting only FUC (F1–F3). The ability of FUC to inhibit the growth of *Candida* was also reported by Oka et al. [53]. There are a number of reports concerning antiviral, antifungal and antibacterial FUC activity. Generally, there are two possible mechanisms that explain its antimicrobial action. Sulfated polysaccharides bind to the bacterial surface, thereby causing damage and nutrients leak. The first hypothesis describes damage of microbial cells by binding sulfated polysaccharides to the bacterial surface causing the leak of nutrients. The second theory explains the antimicrobial activity effect of fucoidans through trapping cationic ions in the medium by negatively charged molecules of sulfated polysaccharides, resulting in a decrease in the bioavailability of nutrients for microorganisms [54].

In all tested strains, a beneficial effect of the GEL addition, both in the placebo and drug-loaded particles, on the antifungal activity was observed (GF1–GF3 versus F1–F3 and GF4–GF6 versus F4–F6). The positive effect of GEL on antifungal activity was also noted by Amit et al. [55]. Designed hydrogels providing peptides with GEL addition ensured their prolonged release (up to 24 h), and thus better antifungal activity on both *Candida albicans* and *Fusarium solani* [55]. Similar conclusions were drawn by the team of Tejada et al. studying the effectiveness of films composed of various polymers (chitosan, carbopol, GEL, arabic gum and alginate) loaded with miconazole nitrate. Their activity was evaluated with using five different *Candida* species (*C. albicans*, *C. parapsilosis*, *C. krusei*, *C. tropicalis* and *C. glabrata*). The best results were noted when as drug carriers, films made of GEL and chitosan were utilized [56]. Additionally, the positive effect of GEL on antifungal activity was confirmed in the case of copper nanoparticles (CuNPs) encapsulated in a gelatin capsule (GEL-CuNPs). The agar diffusion method showed stronger effect of encapsulated nanoparticles (GEL-CuNPs) against both *Fusarium oxysporum* and *Phytophthora parasitica* compared to CuNPs [57].

## 4. Conclusions

In this study, GEL impact on the properties of FUC microparticles prepared by the spray drying was analyzed. It was shown that both pure FUC solutions and solutions containing GEL can be utilized in the spray drying process, and this technique can be applied to formulate spherical FUC and FUC/GEL microparticles. However, the GEL addition slightly reduced the efficiency of the drying process and the percentage of POS loading. GEL presence in microparticles significantly improved swelling capacity, mucodhesiveness and prolonged POS release. Furthermore, all developed microparticles inhibited the growth of *Candida* spp. strains, but formulations with GEL displayed higher antifungal activity. The presented data might be supportive for future investigations over the application of FUC/GEL matrices as multicompartment drug carriers in oral or vaginal administration.

## Figures and Tables

**Table 1:** Composition of designed microparticles.

Formulation	FUC Concentration (%)	GEL Concentration (%)	POS Concentration (%)
F1	10	-	-
F2	15	-	-
F3	20	-	-
F4	10	-	10
F5	15	-	10
F6	20	-	10
GF1	10	5	-
GF2	15	5	-
GF3	20	5	-
GF4	10	5	10
GF5	15	5	10
GF6	20	5	10

**Table 2:** Quality evaluation of FUC (F1–F6) and FUC/GEL (GF1–GF6) microparticles.

Formulation	Percent Loading (%)	Encapsulation Efficiency (%)	Production Yield (%)	Particle Size (µm)	Moisture Content (%)
F1	-	-	67.58 ± 7.37	14.89 ± 3.22	8.23 ± 4.71
F2	-	-	66.88 ± 4.31	16.97 ± 4.89	6.61 ± 2.99
F3	-	-	65.88 ± 4.94	17.91 ± 4.35	9.81 ± 2.84
F4	46.16 ± 0.47	92.33 ± 0.94	68.89 ± 15.12	12.28 ± 6.07	8.82 ± 4.17
F5	43.80 ± 3.31	109.49 ± 9.39	63.21 ± 4.61	12.02 ± 5.14	8.30 ± 4.89
F6	34.91 ± 2.84	105.83 ± 8.17	60.48 ± 3.03	13.07 ± 6.57	8.07 ± 4.16
GF1	-	-	54.04 ± 8.81	13.89 ± 4.93	6.74 ± 2.12
GF2	-	-	47.66 ± 5.79	14.46 ± 6.73	6.34 ± 1.89
GF3	-	-	40.52 ± 3.13	16.03 ± 6.72	8.23 ± 3.24
GF4	46.14 ± 2.32	69.17 ± 3.48	40.60 ± 6.41	12.42 ± 5.58	8.42 ± 3.25
GF5	40.71 ± 1.65	80.19 ± 2.69	49.63 ± 8.05	13.54 ± 6.02	5.19 ± 0.87
GF6	30.97 ± 4.64	72.90 ± 8.93	48.38 ± 8.26	14.96 ± 5.39	6.88 ± 1.93

(mean ± SD, *n* = 3).

**Table 3:** Models of POS release from fucospheres and FUC/GEL microparticles.

Formulation	Zero Order Kinetics		First Order Kinetics		Highuchi Model		Hixson- Crowell Model		Korsmeyer-Peppas Model		
	R^2^	K	R^2^	K	R^2^	K	R^2^	K	R^2^	K	*n*
			**SVF (pH 4.2)**								
F4	0.83	1.17	0.55	0.89	0.81	7.59	0.81	2.65	0.79	0.27	0.09
F5	0.85	1.50	0.64	0.59	0.83	9.94	0.83	2.58	0.77	0.07	0.19
F6	0.96	1.34	0.55	0.32	0.86	9.15	0.76	3.05	0.71	0.05	0.19
GF4	0.74	3.28	0.52	0.05	0.91	21.20	0.99	0.27	0.74	0.27	0.16
GF5	0.71	0.39	0.50	0.06	0.88	21.96	0.99	0.26	0.72	0.27	0.18
GF6	0.82	3.46	0.50	0.06	0.95	21.72	0.99	0.24	0.75	2.56	0.21
			**0.1 M HCl (pH 1.2)**								
F4	0.80	2.91	0.98	0.09	0.94	18.41	0.94	4.25	0.77	0.24	0.19
F5	0.81	2.89	0.99	0.08	0.98	17.73	0.97	4.36	0.84	0.23	0.19
F6	0.85	2.32	0.95	0.05	0.96	14.37	0.92	4.29	0.82	0.21	0.16
GF4	0.82	2.84	0.98	0.07	0.95	17.86	0.95	4.23	0.75	0.24	0.18
GF5	0.99	3.32	0.98	0.08	0.98	18.73	0.99	4.56	0.95	0.18	0.22
GF6	0.99	3.50	0.97	0.09	0.96	20.15	0.99	4.66	0.92	0.16	0.28

R^2^: correlation coefficient, K: release constant, and *n*: the release exponent.

**Table 4:** Antifungal activity of obtained fucospheres and FUC/GEL microparticles.

Formulation	Name of Strain		
	*C. albicans*	*C. parapsilosis*	*C. krusei*
	Zone Inhibition (mm)		
Control *	11.7 ± 1.9	40.7 ± 3.4	30.8 ± 1.1
DMSO	1.9 ± 0.8	0.0 ± 0.0	1.3 ± 0.2
POS/DMSO	10.5 ± 1.3	45.5 ± 2.4	33.5 ± 2.9
F1	3.7 ± 3.2	11.2 ± 0.8	6.1 ± 3.6
F2	5.8 ± 5.3	11.3 ± 2.3	9.2 ± 6.2
F3	7.1 ± 5.7	16.3 ± 1.7	13.8 ± 1.5
F4	29.3 ± 1.1	34.3 ± 1.5	29.5 ± 3.6
F5	24.3 ± 1.2	36.5 ± 2.6	25.7 ± 3.0
F6	22.2 ± 3.3	38.0 ± 1.4	24.9 ± 1.8
GF1	17.3 ± 3.6	31.8 ± 2.1	27.6 ± 2.2
GF2	18.2 ± 1.1	34.8 ± 3.1	21.6 ± 1.7
GF3	14.2 ± 2.4	35.0 ± 1.8	22.3 ± 3.2
GF4	28.7 ± 2.1	43.2 ± 2.5	29.5 ± 3.5
GF5	27.2 ± 1.0	42.6 ± 6.1	29.3 ± 4.1
GF6	27.2 ± 1.3	42.5 ± 3.2	27.9 ± 0.9

(mean ± SD, *n* = 3) * Tablet with fluconazole.

## Data Availability

Data are contained within the article; raw data are available upon request.
